# A Review of Emerging Goose Astrovirus Causing Gout

**DOI:** 10.1155/2022/1635373

**Published:** 2022-08-28

**Authors:** Chenggang Liu, Minhua Sun, Ming Liao

**Affiliations:** ^1^Institute of Animal Health, Guangdong Academy of Agricultural Sciences, Guangzhou 510640, China; ^2^Key Laboratory for Prevention and Control of Avian Influenza and Other Major Poultry Diseases, Ministry of Agriculture and Rural Affairs, Guangzhou 510640, China; ^3^Key Laboratory of Livestock Disease Prevention of Guangdong Province, Guangzhou 510640, China; ^4^Shanwei Academy of Agricultural Sciences, Shanwei 516699, China

## Abstract

In recent years, an infection in geese caused by goose astrovirus (GAstV) has repeatedly occurred in coastal areas of China and rapidly spread to inland provinces. The infection is characterized by joint and visceral gout and is fatal. The disease has caused huge economic losses to China's goose industry. GAstV is a nonenveloped, single-stranded, positive-sense RNA virus. As it is a novel virus, there is no specific classification. Here, we review the current understanding of GAstV. The virus structure, isolation, diagnosis and detection, innate immune regulation, and transmission route are discussed. In addition, since GAstV can cause gout in goslings, the possible role of GAstV in gout formation and uric acid metabolism is discussed. We hope that this review will inform researchers to rapidly develop effective methods to prevent and treat this disease.

## 1. Introduction

**Figure 1:**
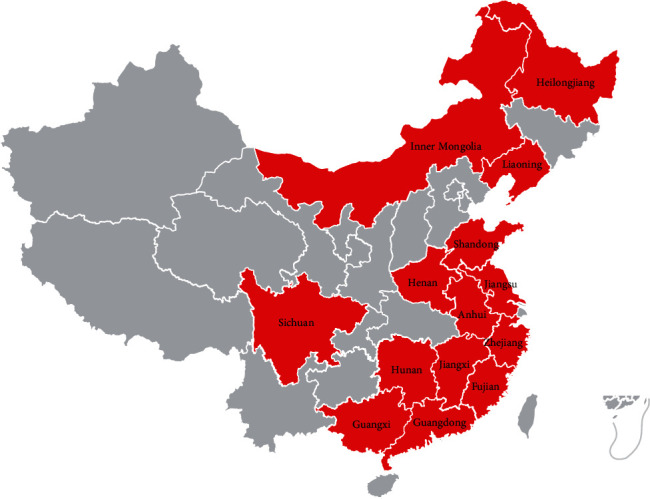
Regions of GAstV infection outbreak in China. The provinces affected with GAstV infection were indicated in red.

Since 2018, an infectious disease with gout as the main symptom has been reported in major goose-producing areas along the southeast coast of China [1–6]. The infection is mainly caused by an astrovirus designated as goose astrovirus (GAstV). The mortality of goslings is over 50%. Within a short time, outbreaks were also reported in inland provinces of China, including Sichuan [7], Inner Mongolia [7], and Heilongjiang [8] (Figure 1).

The disease has caused large economic losses to the goose industry. The infected goslings showed symptoms of depression and anorexia, and the eyelids of some goslings became grey and cloudy [5]. Growth is also inhibited [9, 10]. GAstV replicates in multiple tissues in the body, including heart, liver, spleen, lung, kidney, bursa, thymus, pancreas, brain, proventriculus, and intestine [10]. The virus copy number in the kidney is the highest, followed by the spleen and liver [9, 10]. Autopsy reveals severe symptoms of visceral and joint urate deposition [6]. Hematoxylin-eosin staining reportedly revealed hemorrhage and necrosis of splenocytes in spleen sections, interstitial hemorrhage in kidney sections, tubular necrosis and glomerular swelling, and urate crystals and vacuolar degeneration of hepatocytes in liver sections [4].

Astroviruses infect a wide range of animals, including humans, mammals, and poultry. Astroviruses were first reported in 1975 as causing diarrhea in children [11]. The term astrovirus reflects the obvious star-like structure on the virus surface [12]. Avian astrovirus was first identified in 1965 from diseased ducklings, but it was not officially recognized as an astrovirus until 1984 [13, 14]. Goose astrovirus was discovered relatively late in 2005, as a case of the onset of gout symptoms in goslings [15].

Gout is believed to be caused by excessive consumption of high-protein and high-calcium diets. However, the epidemic has not been improved by replacement with low-protein diets [16, 17]. The real cause of gout in goslings was not determined until researchers isolated a goose-derived astrovirus from the tissue of a diseased goose [1]. The leading cause of death of goslings infected by GAstV is gout. Thus, this paper discusses factors causing gout and abnormal uric acid metabolism in poultry.

## 2. Classification and Structure of Astrovirus

Astroviruses are nonenveloped, single-stranded positive-sense RNA viruses. GAstV is small and round (approximately 30 nm in diameter). Transmission electron microscope reveals stellate protrusions on its surface [18]. The genome consists of a 5′-untranslated region (UTR), three open reading frames (ORFs; ORF1a, ORF1b, and ORF2), a 3′-UTR, and a poly (A) tail [19]. The genome length of astroviruses varies slightly from species to species. The genome length of GAstV is approximately 7.2 kb, including approximately 10 and 200 nt for a 5′-UTR a 3′-UTR, respectively [20]. An overlapping region between ORF1a and ORF1b contains a highly conserved ribosomal frameshifting signal (RFS) sequence (5′-AAAAAAC-3′) and a downstream hairpin structure [5]. RFS is critical to downstream RNA-dependent RNA polymerase (RdRp) translation [21]. ORF1a and ORF1b encode nonstructural viral proteins (NSPs) including transmembrane domains (TMs), serine protease motifs, zinc finger protein model, nuclear localization signal, and RNA-dependent RNA polymerase [20]. ORF2 encodes a viral capsid protein (CP) with the most remarkable diversity of the whole genome, consisting of N-terminal conserved regions and C-terminal highly variable regions. The CP encoded by the highly variable region is distributed on the outer surface of virus particles, forming a capsid spike structure. The capsid spikes are the structural barrier of astrovirus and participate in the recognition of cell surface-related receptors, host immune response, and mediation of cell invasion [22, 23]. The CP encoded by astrovirus is approximately 90 kD (viral protein [VP] 90) and assembles into immature virus particles in cells [24]. VP90 is cleaved by caspase to produce VP70, which is then cleaved by trypsin to VP34, VP27, and VP25 [25]. These three proteins are distributed on the surface of the virus to form mature virus particles [26, 27]. VP34 is encoded by the conserved S domain at the N-terminus of ORF2. VP27 and VP25 are encoded by the P1 and P2 domains, respectively, at the C-terminus of ORF2, which contains neutralizing antibody epitopes and receptor binding domains [28]. VP34 and VP27 determine viral infectivity rather than VP25 [29]. VP34 is folded to form the shell structure of the core region of the virus particles. VP27 and VP25 polymerize to form a dimer structure that comprises the spike structure on the outer surface of the virus particles [30]. Crystal structure analyses of astrovirus CP have focused mainly on human astrovirus [24, 30]. Due to the relatively late discovery of GAstV, no structural protein analysis has been done yet.

Astroviruses are classified by the International Committee on Classification of Viruses into two distinct genera, *Mamastrovirus* (MastV) and *Avastrovirus* (AAstV), according to the natural host. The amino acid sequence of ORF2 has been used as the basis of further classification of astrovirus species. Astroviruses with an average amino acid distance ≤ 0.781 are considered distinct species [31, 32]. The MastV contains 19 recognized astrovirus species (MastV 1-19) that are distributed in two genotypes (GI and GII) [31, 32]. According to the existing classification principles and criteria, Guix et al. [33] divided 14 other unclassified mammalian astroviruses into MastV 20-33. As for the classification of AAstV, there are only three recognized species of avian astrovirus: *Avastrovirus*1 (AAstV-1), *Avastrovirus* 2 (AAstV-2), and *Avastrovirus* 3 (AAstV-3). AAstV-1 is turkey astrovirus 1 (TAstV-1), AAstV-2 includes avian nephritis virus1 and 2 (ANV-1 and ANV-2, respectively), and AAstV-3 contains turkey astrovirus 2 (TAstV-2) and duck astrovirus1 (DAstV-1) [31]. Similar to the situation in MastV, Bosch et al. divided four other unclassified avian astroviruses into AAstV 4-7. AAstV*-*4 is chicken astrovirus (CAstV), AAstV*-*5 is pigeon avian nephritis (Pi-ANV), AAstV*-*6 is wood pigeon astrovirus (WPiAstV), and AAstV*-*7 is feral pigeon astrovirus (FPiAstV) [32].

**Figure 2:**
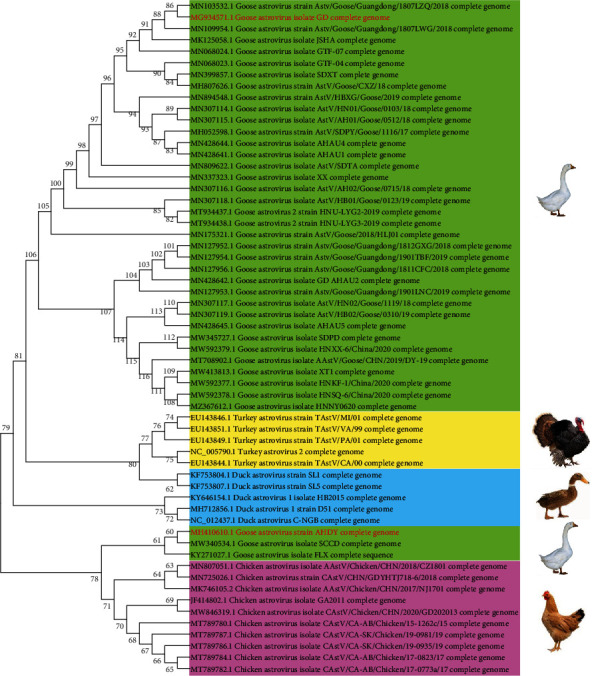
Phylogenetic analysis of the complete astrovirus sequence using MEGA 7.0. The phylogenetic tree was constructed using the neighbor-joining method with 1,000 bootstrap replicates and the composite likelihood model. Each background colour represents an astrovirus species. The red font indicates the representative strains.

GAstV has not yet been systematically classified. A phylogenetic tree analysis of the GAstV strains recently reported in China revealed two groups (Figure 2). The genome nucleotide homology between the two groups is very low. In the AHDY and GD representative strains in the two groups, the homology was as low as 47.68%. However, there was a high level of homology within each group. In the group represented by the GD and AHDY strain, the nucleotide homology of the GAstV genome was ≥97% and ≥98%, respectively. An exception in the latter group was the FLX strain; the genome nucleotide homology of the AHDY and FLX strains was only 89.68%. The difference was mainly in the nucleotide sequence of the ORF2 gene of AHDY and FLX, which displayed only 72.36% homology, while the homology of other genes was 96.77%.

## 3. Separation and Detection of GAstV

The general isolation method of GAstV is to propagate the virus on goose embryos or primary goose kidney cells. The replication ability of GAstV is initially poor. However, after four passages in goose embryos, the virus is 100% lethal to embryos [34, 35]. As there are no specific pathogen-free (SPF) goose or goose embryo models currently, every time the virus is propagated, other pathogens in the goose embryo must be excluded. This makes propagation of the virus very inconvenient. Researchers have attempted to replace goose embryos with SPF chicken or duck embryos in propagating GAstV. Some strains have failed to adapt to chicken and duck embryos. For example, the CXZ18 strain only propagates in goose embryos [18]. Although it is an evolutionary branch of the GD strain, the SDPY strain can establish effective infection in chicken embryos [4]. The SDXT strain can infect duck embryos [36]. Although GAstV can replicate in male white leghorn chicken liver hepatocellular carcinoma cell lines, some strains cause cytopathic effects [37], while others do not [6]. At present, there is no unified laboratory host system, which makes it difficult to study GAstV.

Based on the advantages of rapid polymerase chain reaction (PCR) primer design, molecular diagnostic detection test was rapidly developed. The rapid detection relies on specific primers of GAstV ORF2 gene in the reverse-transcription loop-mediated isothermal amplification (RT-LAMP) assay [38], TaqMan-based one-step real-time RT-PCR assay [39], and SYBR Green I real-time PCR assay [40]. There are also virus detection methods based on GAstV ORF1a [41] and ORF1b [42]. Advantages of all these molecular detection methods include simplicity, rapid performance, sensitivity, and high specificity, which makes them valuable in epidemiological research. An antibody-based detection of ORF2 protein (a vital CP) has also been established. GAstV in goose allantoic fluid and tissue homogenate can be detected by immunochromatography strips based on colloidal gold nanoparticles of ORF2 protein antibody [43]. Recently, several monoclonal antibodies against ORF2 protein have been produced. Epitope mapping revealed that these epitopes are highly conserved in GAstV-1, but not in other AAstVs. One of the monoclonal antibodies can detect GAstV-1, but not GAstV-2, and a sandwich ELISA method was designed to detect GAstV-1 [44]. The establishment of these antibody detection methods can track seroconversion in geese. In an epidemiological investigation, we found that coinfection of GAstV in the two groups represented by the GD and AHDY strains often occurred in livestock farms. Commercial geese can be simultaneously infected with many different viruses or different astrovirus subtypes. Therefore, detection methods need to identify GAstV and must also be able to distinguish between different genotypes. In a recent study, duplex TaqMan real-time RT-PCR was used to distinguish two GAstVs represented by GD and AHDY strains [45]. This refinement makes up for the gaps in detection methods.

## 4. Regulation of Innate Immunity by GAstV

The stellate protrusions of GAstV are formed by the C-terminus of CP encoded by ORF2. These protrusions are the structural barrier of GAstV. The viral CPs wrap viral nucleic acids and also determine cell tropism, mediate virus invasion, and stimulate the host immune response through interaction with the host. In one study, ORF2 efficiently activated the innate immune response and induced a high level of oligoadenylate synthase-like (OASL) *in vivo* and *in vitro*. Interestingly, GAstV restricts its replication by triggering OASL via ORF2 [46]. A truncation assay further revealed that the P2 domain of ORF2 contributed to stimulating OASL, whereas the acidic C-terminus of ORF2 attenuated the activation [46]. There have been relatively few studies on GAstV CP. As antigenic epitopes of GAstV continue to be discovered, the understanding of the function of GAstV CP will deepen.

In addition to activating OASL, GAstV can regulate innate immunity in several ways. GAstV infection induces the activation of pattern recognition receptors that include retinoic acid-inducible gene I (RIG-I), melanoma differentiation-associated gene 5 (MDA-5), and Toll-like receptor 3 (TLR3) and key adaptor molecules, including myeloid differentiation factor 88, mitochondrial antiviral signaling gene, and interferon regulatory factor 7 in the spleen and kidney. Infections also upregulate the gene expression of interferon-gamma (IFN-*α*) in the spleen and the antiviral proteins myxovirus-resistant 1, OASL, and IFN-induced transmembrane protein 3 in the spleen and kidney [47]. Moreover, the expression level of inducible nitric oxide synthase was shown to be very high in the spleen and kidney, and interleukin (IL)-1*β* and IL-8 were also upregulated in the spleen after infection [47]. Another study also confirmed that GAstV could induce RIG-I, MDA-5, and TLR3 overexpression. The authors also reported infection-mediated upregulated expression of IFN-*β*, inflammatory cytokines (IL-8, tumor necrosis factor-alpha), antiviral proteins (Mx, OASL, and double-stranded RNA-dependent protein kinase), and major histocompatibility complex class I [48]. In contrast, the expression of the proinflammatory cytokine IL-6 was inhibited at 3 days postinfection [47, 48].

Considering that the structural protein of GAstV activates the innate immune antiviral genes, these studies offer a possible explanation for why GAstV infection clears spontaneously and clinical symptoms disappear after approximately 14 days. However, the underlying mechanism is not clear and many more studies will be needed to define the immune response mechanism of GAstV infection.

## 5. GAstV Transmission Route and Host

GAstV is naturally spread via the fecal-oral route [49]. Recent evidence suggests that GAstV can also spread vertically [37]. A high prevalence of GAstV was observed in asymptomatic breeding goose flocks, and the nucleic acid of GAstV was detected in either breeder geese or their progeny [37]. After inoculating breeder geese with GAstV, the virus RNA was detected in the vitelline membrane, embryos, and allantoic fluid of goose embryos laid by infected geese. Moreover, the ORF2 gene of GAstV isolated from goose embryos shared almost 100% homology to the virus nucleotide isolated from the goose ovary that produced these goose embryos [37]. Vertical transmission did not affect egg production but did reduce the hatching rate and increase embryo mortality [37]. Once GAstV is prevalent in goose farms, the virus will spread nationwide with the sale of goose eggs. This poses a considerable challenge to epidemic prevention.

The age of infection has a significant impact on GAstV infection [9]. Experimental infections have revealed that GAstV (SDPY strain) is highly pathogenic to goslings aged 1-15 days. The infection leads to growth inhibition, severe visceral urate deposition, and even death. In contrast, the symptoms following infection are mild in goslings aged 25-35 days [9]. The virus copies of geese infected at 1-15 days of age were higher than those of older geese, especially in the vital organs, such as the liver and kidney [9].

There is abundant evidence that astroviruses can cross the species barrier. A highly acute disease characterized by visceral gout was reported in a Muscovy duck farm in Henan Province. After isolation and identification, the virus causing gout symptoms in Muscovy ducklings was revealed to be GAstV (HNNY0620 strain) [50]. Similar diseases have been described in commercial duck farms (SDTA strain) [51] and Cherry Valley ducklings (SDXT strain) [36] in Shandong Province, China. A preliminary viral challenge study demonstrated that 7-day-old chickens inoculated with GAstV could cause clinical visceral gout [52]. Cross-species transmission of GAstV may be due to its mutational and recombination abilities. Like most RNA viruses, GAstV replication lacks proofreading capability, resulting in genetic diversity [53]. Such recombination events create opportunities for cross-species transmission. A recent recombination analysis revealed putative recombination sites in the Jiangsu GAstV strain, which probably originated from GAstV strains in Anhui and Shandong Provinces, accompanied by the recombination of different strains in Anhui Province [54]. Although susceptibility in different species has not been systematically verified by GAstV, as in severe acute respiratory symptom-coronavirus 2 (SARS-CoV2) [55], the presence of TAstV-2 antibodies has been demonstrated in people exposed to turkeys in the United States [56]. The findings highlight the need for close attention to the evolutionary direction of the virus.

GAstV is considered the main pathogen causing gout in goslings. However, according to Koch's postulates, experimental GAstV infection is not very effective in producing gout symptoms, and the incidence of gout is low. One interesting observation is that GAstV is not only detected in goslings with severe gout but is also often accompanied by a mixture of other pathogens [20, 57, 58]. The probable reason is that the gout caused by GAstV is conditional. However, confirmatory experiments need to be done. GAstV nucleic acid can also be detected in goslings without gout symptoms [9]. This finding raises questions about the pathogenic role of specific GAstV strains. In poultry breeding, many factors can induce gout symptoms. Astrovirus infection is a crucial factor. Therefore, this review next explores the possible role of astrovirus in avian gout and its effect on the metabolic disorder of avian uric acid, to find effective methods to prevent and treat gout in goslings.

## 6. Factors Causing Gout in Poultry

Poultry gout, also known as urate deposition or urolithiasis in poultry, refers to the hyperuricemia caused by excessive urate that accumulates in the blood and which cannot be quickly removed from the body. The result is greatly increased uric acid levels in the blood [59]. There are two main factors leading to gout: excessive uric acid in the body and host uric acid excretion disorder [60].

The main factor leading to excessive uric acid production is consumption of a large amount of protein feed rich in nucleoprotein and purine bases, including animal viscera, fish meal, soybean, and other components [61, 62]. Increasing protein in feed, especially nucleoprotein, leads to the formation of progressively more ammonia in the body [63]. Uricemia may occur if the rate of urate production exceeds the excretion capacity of the urinary organs. In one study, a high-protein diet (22% crude protein) in geese resulted in a significant increase of uric acid levels in the blood and formation of urate crystals formed, leading to gout [64].

When inflammation and obstruction occur in the kidney or ureters, uric acid excretion is blocked and urate accumulates in the blood. The urate then deposits on the surface of the pleura, pericardium, peritoneum, mesentery, liver, kidney, spleen, intestine, and other organs [65]. Anything that causes kidney or urinary tract injury, or excessive urine concentration and excretion obstacles, can contribute to uricemia. The factors of uric acid excretion disorder mainly include those causing poultry renal insufficiency: infectious factors involving pathogenic microorganisms that can cause renal function damage and noninfectious factors. The common feature of infectious factors is that they can cause nephritis and renal injury and obstruct the excretion of urates. Infectious factors include kidney-type infectious bronchitis virus [66, 67], inclusions caused by inflammation and egg drop syndrome 76 (EDS-76) of fowl adenovirus [68], renal cryptosporidiosis [69], and, more recently, GAstV [34]. For example, pathogenic microorganisms, such as chicken nephropathogenic infectious bronchitis virus (IBV) and infectious bursal disease virus, can directly damage the renal tissue and cause the disintegration of renal cells [66, 70]. GAstV infection reportedly increases autophagy, destroys intercellular connections of renal tubular epithelial cells, and damages podocytes of kidneys of infected geese [71]. GAstV can also induce lymphocyte apoptosis, reticular fiber destruction, and CD8^+^ T cell depletion in the spleen [72].

Noninfectious factors include nutritional and toxic factors. As an example of a nutritional factor, the lack of vitamin A in feed can cause metabolic disorders of renal tubules and ureteral epithelium, reduce mucus secretion, produce ventilated nephritis, and hinder the excretion of uric acid [61]. At the same time, as an important regulator of immune function, vitamin A has an antiviral role by regulating humoral immunity [73, 74] and cellular immunity [75]. Vitamin A deficiency allows virus to easily enter the host [76–78].

Feed that contains too much calcium causes hypercalcemia after intestinal absorption [79]. The balance of uric acid cannot be maintained in the blood. As a consequence, crystals form in the kidney, resulting in gout and stone symptoms in birds [80]. Several studies reported that when the dietary calcium content of goslings reached 3.1% (approximately 0.9% of normal), the imbalance of the ratio of calcium and phosphorus formed an ectopic deposition of calcium, resulting in poor urination [81–83]. In addition, studies have shown that the virus disrupts the host's electrolyte balance or acid-base balance, leading to nutrient imbalance and death [84–87]. The levels of 1*α*-hydroxylase and vitamin D receptor in lung tissues of mice infected with H9N2 avian influenza virus were reportedly significantly increased, which led to the increased calcium load in the kidney [88]. Excess calcium disrupts the electrolyte balance and acid-base balance of body fluids. Excessive salt, insufficient drinking water, reduced urine volume, and excessive urine concentration can also cause uric acid excretion disorder [67, 82, 83, 89]. An investigation into whether GAstV has similar effects requires further research. In addition to the above factors, from the perspective of animal welfare, the feeding environment is also a factor of causing gout.

## 7. Uric Acid Metabolism in Poultry

A metabolite of proteins is ammonia. Mammals circulate ammonia through ornithine, which is converted into urea by arginase and discharged by the kidneys [90]. Due to the lack of arginase in poultry, the ammonia produced metabolically cannot be synthesized into urea [91]. At the same time, there is no glutamine synthetase in the poultry kidney, and ammonia cannot be carried by glutamine [92]. Therefore, the ammonia protein metabolite can only be excreted in the form of uric acid through purine nucleotide synthesis and a decomposition pathway [93]. The kidney is the place where uric acid is produced in poultry and is the only excretion pathway of uric acid [94, 95]. Therefore, the structure and function of the kidney directly determine whether the metabolism of uric acid in poultry is normal or not.

Purine nucleotides are synthesized in two ways. The first is the *de novo* synthesis of purine nucleotides from simple materials, such as ribose phosphate, amino acids, one carbon unit, and CO_2,_ through a series of enzymatic reactions [96]. Viruses rely on reprogramming of the host metabolism to provide themselves with amino acids required for replication. Examples of viruses are gallid alphaherpesvirus 1 [97] and Newcastle disease virus (NDV) [98]. However, there have been few studies on the effect of GAstV on host metabolism. A metabonomic analysis in our laboratory found that, compared with the plasma of goslings in the control group, all components required for *de novo* synthesis of purine nucleotides in the plasma of goslings killed by gout caused by GAstV infection were highly expressed (data not published). Therefore, systematically exploring the changes of host metabolism in GAstV infection will help to promote new preventive measures from a new perspective.

The second route of purine nucleotide synthesis is to use the free purine or purine nucleoside in the body to synthesize purine nucleotides through a simple reaction process termed the salvage pathway [99]. GAstV, as an RNA virus, produces a large number of nucleic acid fragments during replication. These nucleic acid fragments also provide raw materials for the remedial synthesis pathway.

*De novo* synthesis of purine nucleotides occurs in the cytosol. It is divided into two stages. In the first stage, hypoxanthine nucleotide (IMP) is synthesized. In the second stage, IMP is transformed to adenine nucleotide (AMP) and guanine nucleotide (GMP) [100]. The key enzymes of these two stages are phosphoribosyl pyrophosphate synthetase (PRPS) and phosphoribosyl pyrophosphate amide transferase (PRPPAT) [100, 101]. Both enzymes can be inhibited by the synthetic products IMP, AMP, and GMP [102, 103]. The increase of PRP can promote the activity of PRPPAT and accelerate the production of ribose 5-phosphate [104]. Interestingly, in a preliminary experiment we performed, the mRNA levels of PRPS and PRPPAT in the kidneys of goslings infected with GAstV were significantly upregulated compared with the control group (data not published). Purine nucleotides are purine rings that are gradually synthesized with phosphoribosyl molecules [105]. IMPs are synthesized first and then transformed into AMPs and GMPs [105]. *De novo* synthesis of purine nucleotides is the main source of nucleotides *in vivo*. However, this process needs to consume raw materials, such as amino acids and a large amount of adenosine triphosphate. The host precisely regulates the speed of synthesis, on the one hand, to meet the needs of purine nucleotides for the synthesis of nucleotides. At the same time, “oversupply” is prevented to save the consumption of nutrients and energy. AMP generates hypoxanthine, which is oxidized to xanthine as catalyzed by xanthine oxidase (XO) to finally generate uric acid [106]. GMP generates guanine, which is converted to xanthine and finally uric acid [106]. Catabolism of purine nucleotides *in vivo* is mainly performed in the liver, small intestine, and kidney. XO is the key enzyme in these organs [107, 108]. XO activity was reportedly significantly increased after NDV infection in a study of the therapeutic effect of vitamin E on the oxidative damage of chicken brain and liver attacked by NDV [109]. In addition, nephropathogenic IBV infection resulted in increases in renal XO gene transcription and serum XO activity, leading to hyperuricemia and reduction of antioxidants in the body [107].

The enzymes involved in purine nucleotide salvage synthesis are adenine phosphoribosyl transferase (APRT) and hypoxanthine-guanine phosphoribosyl transferase (HGPRT) [104, 110]. Human cytomegalovirus infection can significantly increase the mutation frequency of the HGPRT gene. However, whether HGPRT protein activity changed was not described [111]. Another study mentioned that butyrate-induced Moloney murine sarcoma virus enhancer promoter element can activate the APRT promoter [112]. Primary gout is caused by the deficiency of purine metabolism-related enzymes, mainly the decreased activity of HGPRT, which limits the remedial synthesis of purine nucleotides but facilitates the production of uric acid. Whether GAstV directly or indirectly regulates these metabolic enzymes in the process of replication has not been reported.

## 8. Prevention, Treatments, and Future Developments

To date, there are no chemotherapeutics, vaccines, or other measures that are fully effective in the control and/or prevention of GAstV infection. Although no stability evaluation of GAstV has been reported, other species of astrovirus are extremely stable and resistant to the inactivation by various disinfectants (chloroform, various detergents, heat, phenols, acidic pH, alcohols, quaternary ammonium salts, and lipid solvents) [113]. Stringent biosafety controls may reduce the likelihood of GAstV infection. In one study, recombinant GAstV CP was inserted into an attenuated goose-origin NDV strain to prepare a bivalent vaccine. Although this vaccine protected against pathogenic GAstV challenge and velogenic NDV challenge, it has not been widely used [114]. However, given the mixed infection between various GAstV subtypes and GPV [57], using other viruses as vaccine vectors may be worthy of further investigation. Before the outbreak of GAstV, the main influencing factor of gout was diet. A high calcium level, low phosphorus level, and vitamin A deficiency in feed can lead to gout [115, 116]. A high-fat diet can lead to lipid metabolism disorders in the host and subsequently increase serum uric acid levels significantly, leading to gout [117]. Gout can also be induced by damp conditions, dark rearing environment, high density of rearing, and insufficient animal activity. Therefore, the feeding environment and the density of geese should be strictly controlled.

In the past few decades, advances in DNA recombination and reverse genetics have dramatically changed the landscape of vaccine development. Furthermore, advances in the transcriptome, proteome, and many other omics have provided powerful tools for the global identification of protective antigens. Although DNA vaccine vector and liposome nanomaterial vector technologies have developed rapidly in recent years, their use in the prevention and treatment of avian diseases is not likely soon due to their low efficiency and high costs [118]. With the increasing understanding of the avian immune system, live bacterial vaccine vectors and viral vaccine vectors that are safe and inexpensive will be developed and applied to the prevention of GAstV. These two vaccine vectors have been valuable in the development of avian adenovirus vaccine [119, 120].

GAstV is an important virus that has been neglected for a long time. Even in the past decade, the view was that the cause of gout in goslings is feeding high-protein feed [15–17]. Further research and technological progress have identified GAstV as the main cause of gout symptoms in goslings [1]. However, the treatment of the disease by veterinarians is limited to eliminating the symptoms of gout, and prevention strategies are based on the previous understanding of gout. Viral infection can cause host metabolic reprogramming [97, 98, 121], which leads to gout symptoms. Treatment addressing metabolism alleviates gout symptoms but has no effect on eliminating GAstV and preventing infection. Effective vaccine design requires the preparation of prophylactic and therapeutic vaccines that mimic the natural process of viral infection. These efforts are based on an understanding of the characteristics of viral transmission and replication. Future studies that address a series of viral infection mechanisms, such as GAstV invasion of host and replication, could fundamentally solve the problem of gout caused by GAstV.
